# Second systolic peak in fetal middle cerebral artery Doppler after intrauterine transfusion

**DOI:** 10.1007/s00404-022-06517-0

**Published:** 2022-03-29

**Authors:** Ladina Vonzun, Nicole Ochsenbein-Kölble, Dalia Balsyte, Roland Zimmermann, Markus Gonser

**Affiliations:** 1 Department of Obstetrics, University Hospital Zurich, Frauenklinikstrasse 10, 8006 Zurich, Switzerland; 2 University of Zurich, Zurich, Switzerland; 3 Department of Obstetrics and Prenatal Medicine, Helios-HSK Kliniken Wiesbaden, Wiesbaden, Germany

**Keywords:** Second systolic peak, Middle cerebral artery, Doppler, Intrauterine transfusion, Pulse wave reflection, Hemodynamic principles

## Abstract

**Objective:**

To evaluate functional relationship between fetal circulatory response to intrauterine transfusion (IUT) as a circulatory challenge and appearance of second systolic peak (P2) in middle cerebral artery (MCA) based on hemodynamic principles.

**Methods:**

According to the concept of pulse wave (PW) propagation and reflection in adults, PWs arrive twice at cerebral circulation, as primary wave caused by left ventricle ejection and secondary after reflection in peripheral arteries. Thus adults show a biphasic contour of systolic blood flow in cerebral arteries. Similar waveforms may appear in fetal MCA-Doppler, as a response to IUT as a circulatory challenge. This is a proof-of-principle study, applying classical hemodynamic principles to fetal circulation. Accordingly, appearance of MCA-P2 may indicate vasoconstriction with increased PW reflection and timing of P2(Δ*t*) should agree with the additional PW travel time down to reflection and return (Tr). To test this agreement, we searched our database for IUTs performed for severe fetal anemia, and compared Δ*t*, obtained by Doppler, with Tr, obtained by hemodynamic calculation using human fetal data. Level of agreement was assessed using Bland–Altman-Plots.

**Results:**

We identified 21 fetuses with adequate Doppler quality for Δ*t* evaluation. In four cases (19%) MCA-P2 was observed before the intervention, and in 17 interventions (81%) thereafter; a highly significant association between IUT and P2 appearance (*p* < 0.001). In these 17 interventions good agreement of P2 timing was found between Doppler assessment: Δ*t* = 80 ± 8 ms, and hemodynamic calculation: Tr = 76 ± 4 ms.

**Conclusion:**

P2 appearance in fetal MCA-Doppler seems to indicate PW reflection due to increased vasoconstriction after IUT. Thus hemodynamic considerations might enable Doppler monitoring of fetal vasoconstriction.

## Introduction

A transient second systolic peak (P2) may appear in fetal middle cerebral artery (MCA) Doppler waveforms after intrauterine transfusion (IUT). In a classical paper on fetal cerebral arteries Doppler waveforms before and after IUT published in 1990 Mari et al. showed cerebral and especially MCA Doppler waveforms with a second systolic peak (MCA-P2), one in a fetus with severe anemia (Hb 4 g/dl), and another one 2 h after IUT [1]. However, in the detailed hemodynamic discussion this particular systolic waveform feature was not mentioned.

Fetal condition after IUT is transiently worsened as the transfused blood has very low pH (< 7.0) and high hematocrit (Hct). Both these conditions increase systematic vasoconstriction in animal models [2–4]. Recently evidence was found that appearance of a second systolic peak in MCA Doppler of human fetuses with severe anemia may indicate increased pulse wave (PW) reflection, with secondary transmission to head and cerebral circulation [5]. Furthermore, observed timing of reflection seems to minimize pulsatile energy consumption [6–8].

We speculate that IUT is associated with higher reflective conditions due to transiently increased fetal vasoconstriction. The aim of our study was to evaluate the functional relationship between the fetal circulatory response to the IUT and the appearance of a MCA-P2 based on a hemodynamic principles.

## Patients and methods

This is a proof-of-principle study applying a classical hemodynamic principles to the fetal circulation.

### The pulse wave model of hemodynamics

In hemodynamics the concept of PW propagation and reflection is well established: cardiac contraction generates a PW and accelerates blood flow. During propagation of the PW to the periphery, reflections in the arterial system occur. Apparently major reflected waves merge to a coherent reflected wave (RW), travelling back with an average velocity, c(Ao), along the aorta (Ao), and finally arriving at the left ventricle (LV) like an echo, after a definite time of return, the so-called reflection time, Tr [9–11].

**Fig. 1:**
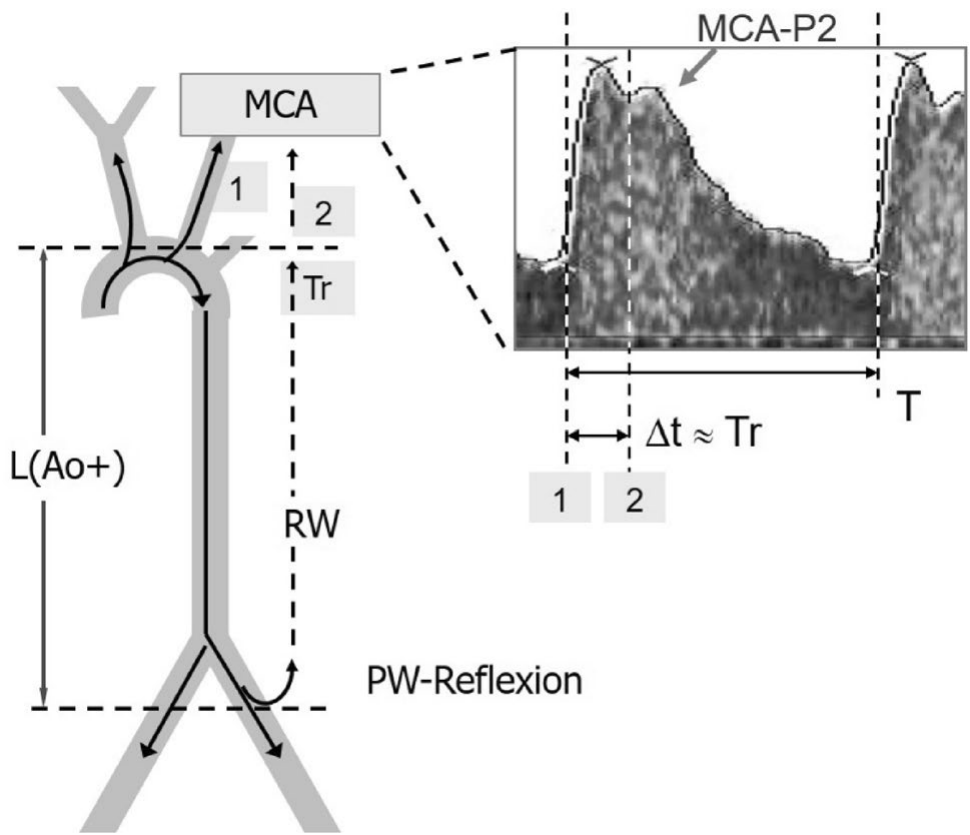
Hemodynamic model. Left: diagram of pulse wave propagation, reflection and cerebral transmission. Right: middle cerebral artery (MCA) Doppler waveform with the time interval Δ*t* to MCA-P2 onset and reflection timing: Tr/*T* ≈ 0.2. Pulse waves reach twice to MCA: by direct transmission [1], and after reflection, return and subsequent transmission [2]. The time interval Δ*t* from MCA waveform onset to MCA-P2 onset corresponds to the reflection time Tr, i.e. the two-way travel time needed for travelling down to reflection and back

Based on this reflection time Tr (i.e. the two-way travel time taken for travelling down to reflection and back (shown in Fig. 1)) and the average PW velocity in the aorta, c(Ao), the functional distance L to reflection may be obtained:

1 $$L \, = \, c\left( {Ao} \right) \cdot {\text{Tr}}/2.$$

This is the concept of the "effective length" *L* of the central arterial system in hemodynamics [12] and corresponds approximately to the path lengths of aorta plus common iliac artery (CIA) as anatomical surrogates, conveniently written as: *L*(Ao +)

2 $$L \, = \, L\left( {{\text{Ao}} + } \right) \, = \, L\left( {{\text{Ao}}} \right) \, + \, L\left( {{\text{CIA}}} \right) \approx L\left( {{\text{Ao}}} \right) + 10\% .$$

Consequently, RW seems to return from a functional distance *L*(Ao +) corresponding to the pelvic region [6, 10, 12] and may arrive at the LV during ongoing systole [11].

After substituting "*L*" by "*L*(Ao +)" in Eq. 1, and rearranging, the following relation for Tr is obtained:

3 $${\text{Tr}} = 2L\left( {{\text{Ao}} + } \right)/c\left( {{\text{Ao}}} \right).$$

Like an echo time, the reflection time Tr is given by the two-way travel distance to reflection and back: 2∙L(Ao +), divided by the average PW velocity in the aorta, *c*(Ao). This relation is the key for the transfer of the classical PW model to fetal circulation.

At the level of the aortic arch, a fraction of the reflected wave RW is diverted cranially to the cerebral circulation as a forward wave, to create the second systolic peak, P2 [13, 14]. Hence this second wave arrives at the cerebral circulation after a temporal delay corresponding to the additional travel time down to reflection and back, and thus is given by the aforementioned reflection time, Tr [15–17].

### Transfer of the pulse wave model to the fetal circulation

If we apply the same principles of PW propagation and reflection to the fetal circulation, then PWs should arrive twice at cerebral circulation too: first by direct transmission, and second after downstream propagation, reflection and subsequent transmission. This second wave accelerates ongoing flow and may create a second systolic peak in fetal cerebral waveforms, if PW reflection is strong enough. By analogy, the interval Δ*t* until P2 onset (contour inflection point) on the fetal MCA waveform should coincide with the temporal delay of reflected PW arrival at the Doppler recording site, given by the reflection time, Tr. To our knowledge, reflection time is not known in the human fetus, but may approximately be calculated with [Eq. 3]: Tr = 2*L*(Ao +)/*c*(Ao).

### Patients

**Fig. 2:**
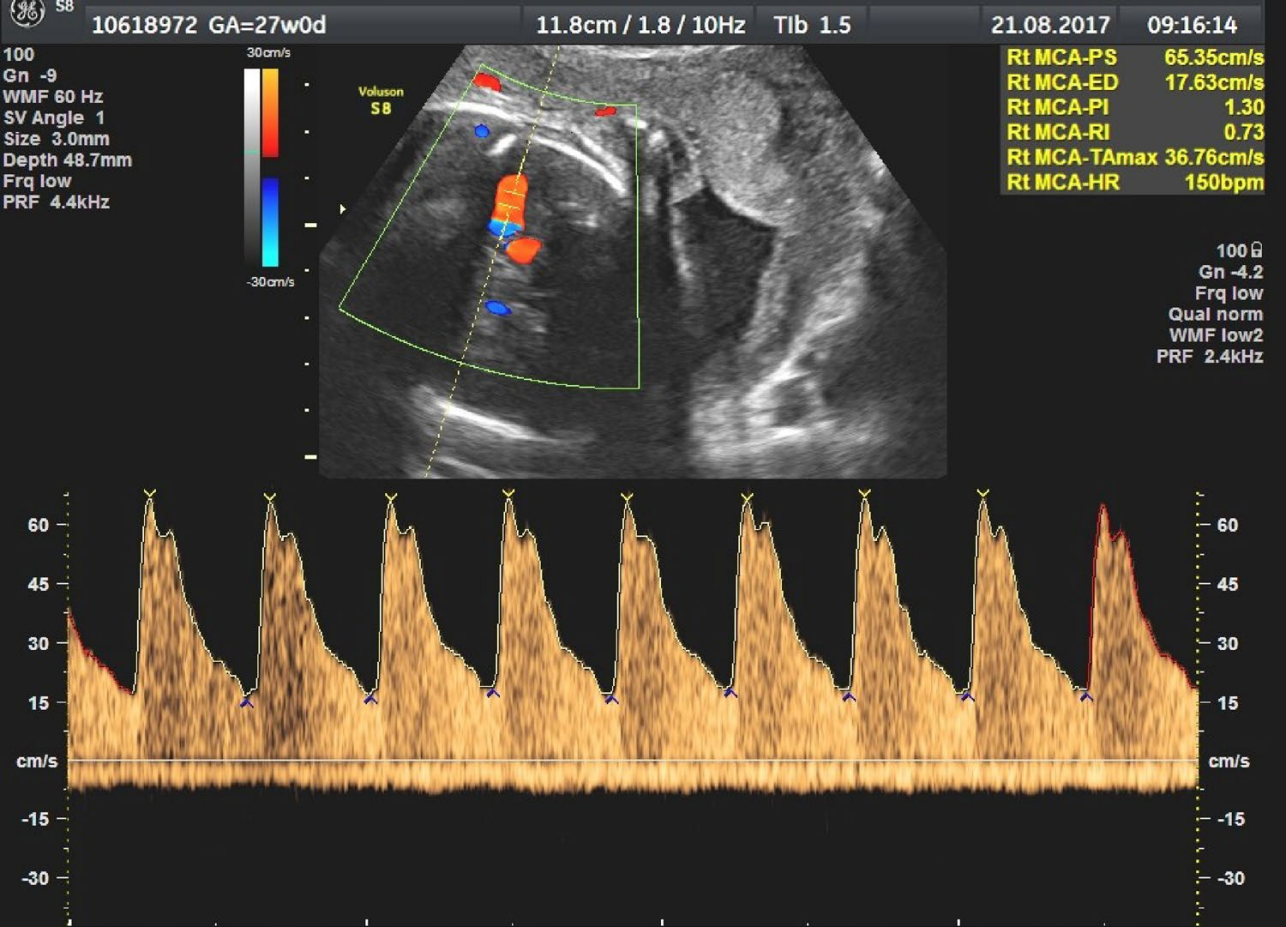
Example of fetal middle cerebral artery (MCA) Doppler with a good image quality presenting a second systolic peak (MCA-P2)

In order to test the validity of the PW model in the fetal arterial system, IUT was chosen as an exemplary, reproducible, extreme fetal distress situation. We performed a preliminary search in our perinatal database for IUTs performed from 2006 to 2018 and reviewed the MCA Doppler waveforms obtained directly before and after IUT for appearance of MCA-P2. All cases with IUT performed for severe fetal anemia (fHb ≤ 0.55 MOM) with a transfusion coefficient ≥ 0.03 (IUT-volume [ml]/estimated fetal weight [g]) [18], and gestational age (GA) between 26 + 0 and 30 + 6 weeks were included if Doppler image quality was adequate for Δt evaluation (shown in Fig. 2).

The time interval (Δ*t*) between MCA waveform onset and MCA-P2 onset was measured as shown in Fig. 1. Reflection time Tr was calculated according to Eq. 3 of the PW model: Tr = 2*L*(Ao +)/*c*(Ao) (shown in Fig. 1). The required factors: *L*(Ao +) = *L*(A0) + *L*(CIA), the anatomical surrogate of fetal functional distance to reflection, and *c*(Ao), the average PW velocity in the fetal aorta, were obtained based on human fetal data as follows:

- For both anatomical parameters, *L*(Ao) and *L*(CIA), GA-adjusted values had been published, and the sum of the vascular lengths was calculated according to the formulas proposed by Szpinda et al. for fetal Ao [19] and CIA [20], yielding: *L*(fAo +) = *L*(Ao) + *L*(CIA)[mm] = 5.242∙GA(wks) − 48.36.
- Similarly GA-adjusted values for the PW velocity in the fetal Ao, *c*(Ao), were obtained according the following formula: c(Ao) [cm/s] = 4.7∙GA(wks) + 125.1 [21]. In this study, *c*(Ao) was assessed from the longitudinal section of the descending fetal aorta at the level of the diaphragm.

The results of the Doppler-recorded time intervals Δ*t*, were compared to the results of the model-calculated PW reflection time, Tr. Data coding and descriptive statistics were performed with SPSS version 26.0 (IBM, SPSS Inc., USA). To evaluate the agreement of the results obtained by both methods, Doppler assessment, Δ*t*, and model calculation, Tr, a Bland–Altman (mean-difference or limits of agreement) plot was used. The intermethod average (Av) of each pair of results: Av(Δ*t*, Tr) = (Δ*t* + Tr)/2, was plotted against the intermethod difference: Diff(Δ*t*, Tr) = (Δ*t* − Tr), and the 95% limits of agreement (LA) between the results of both methods, Δ*t* and Tr, were calculated.

The study was conducted in accordance with the approval of the local Ethics Commission (KEK-ZH. Nr.2020–01225).

## Results

We could identify 21 interventions meeting all inclusion criteria and adequate Doppler quality for Δ*t* evaluation. In 4 cases (19%) MCA-P2 was observed before the intervention, and in 17 interventions (81%) MCA-P2 was observed thereafter, thus indicating a highly significant association between IUT performed for severe anemia and observation of MCA-P2 (*p* < 0.001, Fisher’s exact test) after the intervention.

Thus 17 interventions remained for Δ*t* measurements and these were performed on 14 fetuses (3 fetuses with repeated interventions). The underlying cause for anemia was maternal red cell alloimmunization in 10 cases, anti-Kell antibodies in 3 cases and fetal parvovirus B12 infection in one case. Mean GA (± SD) at IUT was 28 ± 1 weeks at a fetal weight of 1061 ± 358 g. Fetal hemoglobin measurements before IUT were 5.6 ± 1.4 g/l followed by IUT with a mean IUT-Volume of 42 ± 12 ml. Neonatal outcome data are presented in Table 1.

**Table 1:** Neonatal outcome data

Nr	GA at birth	Mode of birth	Birth weight	Apgar	pH	Hct (%)	Neonatal therapy
1	37 + 3	Vaginal	3170	6–9–9	7.26	48	None
2	30 + 5	C-Section	1450	2–9–9	7.33	20	Transfusion
3	*						
4	*						
5	*						
6	*						
7	37 + 5	Vaginal	3030	8–9–9	7.15	43	None
8	37 + 1	Vaginal	2850	8–9–9	7.26	55	None
9	37 + 1	Vaginal	2750	8–9–9	7.31	59	None
10	36 + 2	C-Section	3040	8–9–10	7.39	51	Phototherapy
11	37 + 3	C-Section	2900	8–9–10	7.38	36	Phototherapy
12	25 + 1	#Vaginal	1120	1–1–1			
13	38 + 3	Vaginal	3060	9–9–10	7.39	42	None
14	37 + 5	Vaginal	2860	8–9–10	7.28	59	None

*Last US at our center took place after 34 + 0 gestational age (GA), women were sent to the hospital close to home for delivery. Outcome data are not available

^#^Progressive Hydrops fetalis despite transfusions. Interdisciplinary decision with the parents for palliative care

The mean value of the time intervals Δ*t*, measured between MCA waveform onset and MCA-P2 onset in Doppler waveforms obtained in these cases after IUT was: Δ*t* = 80 ± 8 ms.

The mean of the vascular path lengths obtained according Eq. 2: *L*(Ao +) = *L*(Ao) + *L*(CIA) using individual, GA-adjusted human fetal data on Ao and CIA lengths was: *L*(Ao +) = 9.7 ± 0.7 cm, and the mean of the aortic PW velocities obtained from individual, GA-adjusted fetal aortic velocity data [21] was: *c*(Ao) = 255 ± 6 cm/s.

Finally, we calculated the individual reflection times Tr according to Eq. 3: Tr = 2∙*L*(Ao +)/*c*(Ao), and obtained as mean value: Tr = 76 ± 4 ms (Table 2).

**Table 2:** Gestational age (GA) adjusted, individual values for: functional distance to reflection, *L*(Ao +) = *L*(Ao) + *L*(CIA), average fetal aortic pulse wave velocity, *c*(Ao), and individually calculated reflection times, Tr = 2*L*(Ao +)/*c*(Ao) and time interval Δ*t* to MCA-P2 onset of 17 IUT procedures

Nr	GA	*L*(Ao +) [cm]	*c*(Ao) [cm/s]	Tr [ms]	Δ*t* (ms)
1	26 + 3	8.8	247	71	76
2	26 + 5	8.8	247	71	87
3	26 + 5	8.8	247	71	83
4	27 + 1	9.3	252	74	86
5	27 + 1	9.3	252	74	67
6	27 + 3	9.3	252	74	71
7	27 + 4	9.3	252	74	84
8	27 + 4	9.3	252	74	71
9	27 + 4	9.3	252	74	84
10	27 + 4	9.3	252	74	80
11	28 + 1	9.8	257	76	83
12	28 + 6	9.8	257	76	79
13	29 + 3	10.4	262	80	88
14	29 + 4	10.4	262	80	79
15	29 + 5	10.4	262	80	95
16	30 + 1	10.9	266	82	83
17	30 + 6	10.9	266	82	67
Mean	28 ± 1	9.7 ± 0.7	255 ± 6	76 ± 4	80 ± 8

**Fig. 3:**
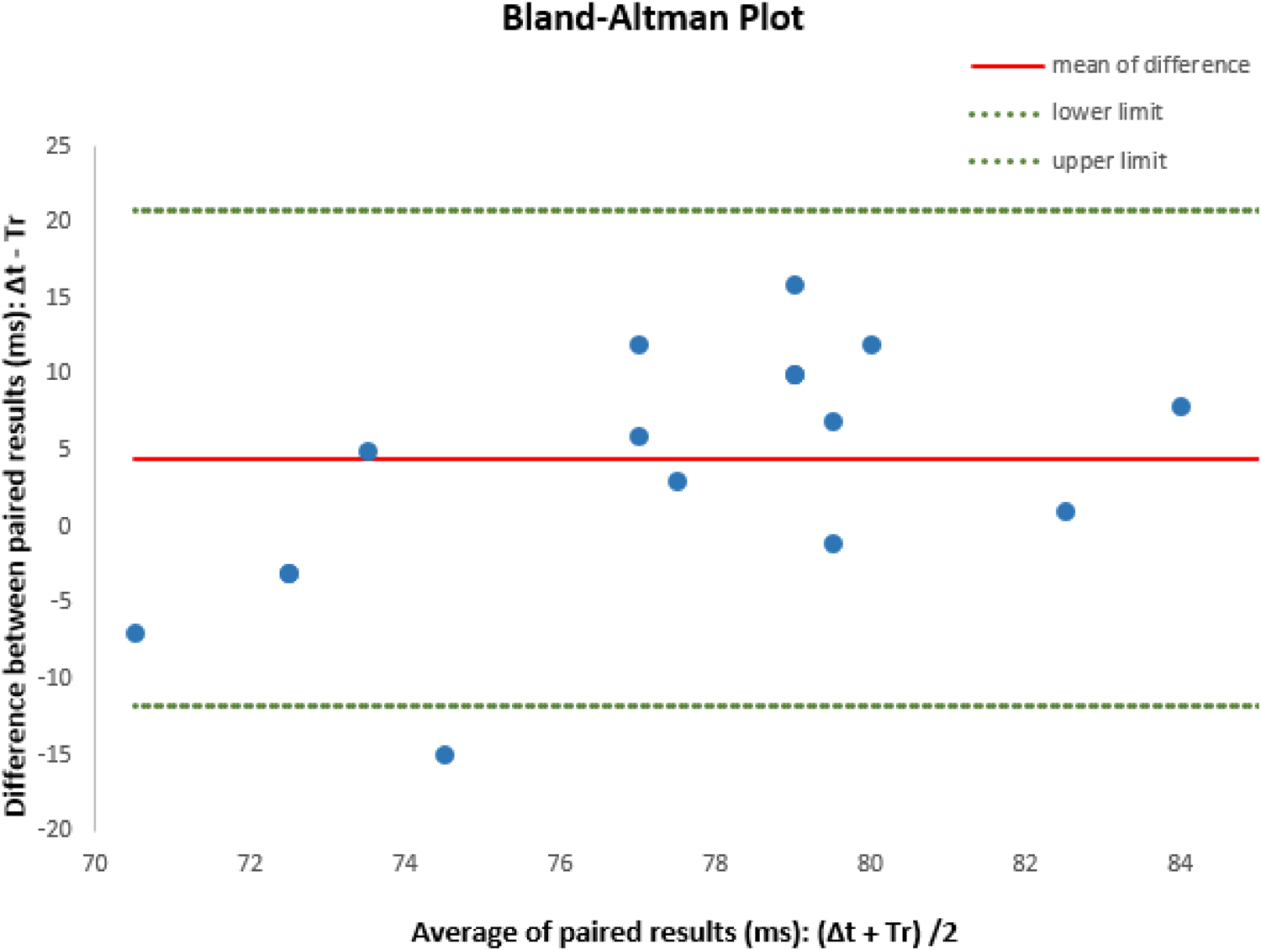
Bland–Altman plot to assess the agreement between Δ*t* and Tr: the intermethod difference between mean Δ*t* (80 ms) and mean Tr (76 ms) was 4.8 ms, and the 95% limits of agreement (LA, ms) were [− 11.8; + 20.8]

Good agreement between the results of both methods, Tr = 76 ± 4 ms and Δ*t* = 80 ± 8 ms, was confirmed by the Bland–Altman plot: the intermethod difference between mean Δ*t* (80 ms) and mean Tr (76 ms) was 4.8 ms, and the 95% limits of agreement (LA, ms) were [− 11.8; + 20.8] (shown in Fig. 3).

## Discussion

This study provides evidence that a second systolic peak, P2, in MCA Doppler might indicate PW reflection due to increased fetal vasoconstriction after IUT. Accordingly, hemodynamic principles might enable Doppler monitoring of fetal vasoconstriction.

MCA Doppler has doubtlessly established its priority role in the diagnosis of fetal anemia [22–24]. In pregnancies complicated by maternal red cell alloimmunization, monitoring the MCA peak systolic blood flow velocity by Doppler assessment was shown to be reliable enough to replace invasive testing. The present study however, directs the focus to an additional, new tool of the MCA Doppler imaging, the second systolic peak, MCA-P2, which may identify increased fetal PW reflection as a marker of fetal distress. There are a number of aspects that deserve detailed consideration.

Fetal MCA Doppler waveforms mainly show a slightly convex [25] systolic downslope, sometimes changing from a more or less discernable systolic shoulder (MCA-S) to a clearly visible but less frequent second systolic peak (MCA-P2). This latter waveform pattern may appear in cerebral and especially in MCA Doppler waveforms after IUT, as shown in the classical paper by Mari [1] and occasionally in fetal MCA waveforms published by others [26–28]. We could observe that MCA-P2 was more frequently persisting in severe anemic fetuses after IUT with a significant transfusion volume, and we decided to study this so far unaddressed or overlooked phenomenon.

IUT performed as therapeutic intervention may transiently worsen the fetal condition due to low pH and high Hct of the transfused blood. Animal models revealed signs of systemic fetal vasoconstriction due to transient acidemia and hyperviscosity after IUT [2–4]. Thus, IUT constitutes a well-defined and quantifiable circulatory stress to the fetal cardiovascular system. In the human fetus IUT was found to increase blood pressure (BP) in the umbilical vein (UV) [29], but we could not localize any report on arterial BP measurements. Whereas in fetal lambs IUT was reported to increase arterial BP [30]. However, in the human fetus IUT was associated with a significant increase in Arginine vasopressin (AVP) levels [31], an antidiuretic hormone and potent vasoconstrictor, which increases peripheral vascular resistance and raises arterial BP in adults [32]. This supports the assumption that IUT may increase vasoconstriction in the human fetus too. According to our preliminary observations, major IUT challenge might be detected by the appearance of MCA-P2.

We looked for corresponding Doppler signs seen in cerebrovascular waveforms in adults, and found that similar systolic waveform modulations were attributed to PW propagation, reflection and cranial transmission [13, 15, 17, 33, 34]. Accordingly, systemic vasoconstriction modulates PW reflection and the shape of cerebrovascular waveforms [12, 13, 35]. Thus, we assumed that this hemodynamic approach might be applicable in fetuses too and be useful to describe the functional relationship between IUT as a circulatory challenge and MCA-P2 appearance as a result of PW reflection. A valid model should enable to describe the response of the fetal arterial system in a predictive way and this is what we could confirm with this study. The temporal delay Tr of reflected wave arrival to MCA, as calculated by the model: Tr = 2*L*(Ao +)/*c*(Ao), coincided with the clinically observed temporal delay Δ*t* to MCA-P2 onset, the assumed sign of fetal PW reflection appearing in MCA Doppler waveforms. Thus, the result of the model-calculated PW reflection time, Tr = 76 ± 4 ms, is comparable to the results of the Doppler-recorded time interval Δ*t* = 80 ± 8 ms. The Bland–Altman method confirmed good agreement between both results (shown in Fig. 3): within the 95% limits of agreement (LA) [− 11.8; + 20.8] (ms), indicating a mid-systolic event in fetuses between 26 and 32 weeks’ gestation, were mean ejection time (systole) was reported between 165 and 175 ms [36–38]. Finally the intermethod difference of 4.8 ms between mean Δ*t* (= 80 ms) and mean Tr (= 76 m) is within the temporal resolution capacity of the Doppler method (~ 10 ms) [39].

The validity of the PW model in the human fetus is supported by recently published studies on optimal pulsatile timing in the mammalian cardiovascular system [6, 40]. Accordingly, energy consumption of pulsatile cardiac action is optimized when the (one-way) transit time to PW reflection takes about 10%, or equivalent, when the (2-way) transit time to PW reflection and return (= Tr) takes about 20% of the cardiac cycle *T*: Tr/*T* ≈ 0.2 [6–8]. Assuming a mean fetal heart rate of 150 bpm (i.e. cardiac cycle *T* = 400 ms), then with our study result: Tr = 76 ms, follows: Tr/*T* = 76 ms/400 ms = 0.19. This indicates nearly optimal pulsatile timing and seems to confirm the validity of the same PW optimization criterion in the fetus. Moreover, a simple look to the MCA waveform pattern allows a brief visual check of this 20% criterion (Tr/*T* ≈ 0.2, Fig. 1).

For ethical and clinical reasons we could not have direct access to the arterial system of the fetus, the subject of our study. This is a main limitation of our study, i.e. we could not measure vasoconstriction or arterial blood pressure to study the fetal circulatory response to IUT directly. Nevertheless, we could observe critical signs of PW reflection and these observations are based on a well-stablished hemodynamic model in (adult) physiology [10, 12, 13, 15, 33–35].

## Conclusion

The transfer of the hemodynamic model of PW propagation and reflection to the fetus is feasible and provides a new, non-invasive access to the fetal cardiovascular system. Accordingly a second systolic peak in fetal MCA Doppler imaging, MCA-P2, seems to indicate increased fetal PW reflection, and may appear in particular circumstances after IUT, a therapeutic intervention associated with transient fetal circulatory distress. Thus consideration of a secondary systolic peak P2 in fetal MCA-Doppler waveforms might open a diagnostic window to observe signs of fetal vasoconstriction after IUT. Whether monitoring of this particular Doppler sign might be of clinical benefit in fetal treatment, remains to be evaluated in systematic clinical studies. Clearly, these hemodynamic considerations provide additional insight and may help to design such a study.

This hemodynamic model approach may also open a window to evaluate transplacental effects of vasoactive substances on fetal cardiovascular system, including anesthetics, tocolytics and antihypertensives [41]. In the meanwhile, the same hemodynamic principle of PW reflection from the arterial system and transmission to cerebral arteries became also the basis for the interpretation of maternal ophthalmic artery Doppler waveforms to predict and to monitor preeclampsia [42–44].
